# De novo autoimmune hepatitis following liver transplantation for primary biliary cirrhosis: an unusual cause of late grafts dysfunction

**DOI:** 10.11604/pamj.2015.21.2.6789

**Published:** 2015-05-04

**Authors:** Rym Ennaifer, Hend Ayadi, Haifa Romdhane, Meriem Cheikh, Hafedh Mestiri, Taher Khalfallah, Najet Bel Hadj

**Affiliations:** 1 Department of Hepato-Gastro-Enterology, Mongi Slim Hospital, Tunis, Tunisia; 2 University of Tunis El Manar, Faculty of Medicine, Tunis, Tunisia; 3 Departement of Surgery, Mongi Slim Hospital, Tunis, Tunisia

**Keywords:** Primary biliary cirrhosis, liver transplantation, de novo autoimmune hepatitis

## Abstract

De novo autoimmune hepatitis (AIH) is a rare disorder first described in 1998. It occurs in patients who underwent liver transplantation for a different etiology. We present the case of a 56-year-old woman who was diagnosed with primary biliary cirrhosis and had liver transplantation for refractory pruritis. Seven years after transplantation, she presented alterations in the hepatic profile with hypertransaminasemia, elevated alkaline phosphatase and gamma-glutamyl-transferase. Her liver functions test also showed elevated IgG levels. Serum autoantibodies were negative except for antimitochondrial antibodies. Histological findings indicated features of AIH without bile duct damage or loss. She had a pretreatment AIH score of 13 points and a post treatment score of 15 points according to the International AIH Group. The patient was treated effectively with prednisolone and her liver function and globulin levels rapidly returned to normal.

## Introduction

Liver transplantation (LT) is a standard therapeutic approach for end-stage acute and chronic liver disease. Approximately 90% of liver transplant patients are alive after 1 year and 75% after 5 years. However, transplant recipients can experience various complications such as acute or chronic rejection, recurrence of primary disease, chronic hepatitis, graft fibrosis and more recently, de novo AIH [1]. Post-transplant de novo AIH connotes the development of AIH after LT for disease other than AIH. It was first described in 1998 and since then subsequent studies have confirmed the occurrence of de novo autoimmune hepatitis in 1-7% of patients from 0 to 9 years after liver transplant [2, 3]. We report the case of de novo AIH after LT for primary biliary cirrhosis (PBC). Etiology, pathogenesis as well as diagnostic and treatment strategies are briefly but comprehensively discussed.

## Patient and observation

A 34 years old woman was diagnosed in 1991 with PBC. The diagnosis was established on 3 criteria: elevation of γ-glutamyl transferase (GGT) and alkaline phosphatase (APL), presence of antimitochondrial antibodies and histological findings on liver biopsy. In 2005, the patient underwent an orthotropic LT for rebellious itch to medical treatment. She received immunosuppressive therapy with prednisolone, tacrolimus, and mycophenolate. Histology of her explanted liver showed features typical of PBC with non suppurative cholangitis, paucity of bile ducts and annular fibrosis. There were no lesions to suggest any overlap with AIH. Postoperative evolution was generally satisfactory. She never experienced an episode of rejection. Seven years after transplantation, she displayed alterations of hepatic profile: ALT increased to 270 U/L (normal range (NR) =40), AST increased to 200 U/L (NR = 40), APL increased to 180 U/l (NR = 130) and γ-GT increased to 50 U/L (NR = 35) without elevation of bilirubin. Ultrasonography using Doppler, cholangiography and liver computed tomography were normal, excluding biliary or vascular complications. Viral hepatitis A, B and C markers were negative as well as IgM antibodies for EBV, CMV and HSV. Tests for anti-nuclear antibody (AAN), antibody to liver/kidney microsomes type 1, smooth muscle antibody (SMA) and SLA/LP autoantibodies were all negative whereas IgG level were increased up to 2 times upper limit of normal. Antimitochondrial antibodies were positive at a level > 1/80. At the time of presentation, she did not receive any medication except immunosuppressive therapy which included tacrolimus (2 milligrams twice a day), with an average trough blood level of 7.2 ng/mL and mycophenolate (2 grammes a day). A liver biopsy was performed and the pathological examination showed a portal and periportal hepatitis with lymphocytes and plasma cells disrupting the limiting plate. There was bridging centrilobular necrosis without duct damage or loss. These finding were suggestive of de novo AIH. The international AIH Group (IAIHG) score was 13, thus falling into the category of probable AIH. The HLA DR3 and DR4 have not been sought and have not been taken into account for the calculation of this score. Prednisone was started at a dose of 0.5 mg per Kg per day. The patient responded rapidly with normalization of aminotransferases at week 4 and a decrease of cholestasis. At week 8 of treatment, the patient suddenly interrupted corticosteroids. Her liver function again was abnormal with an increase of aminotransferase to 4 times normal. The steroids were reintroduced at the same dose allowing rapid normalization of aminotransferases. Using the scoring system of IAIHG, the patient had probable AIH (pretreatment score 13, post treatment score 15).

## Discussion

We have described the case of de novo AIH after LT for PBC. This form of graft dysfunction occurs among patients who have undergone LT for other reason than AIH but remain a rare disorder among patients transplanted for PBC. Indeed, these patients are rather at risk for PBC recurrence in up to 20% of cases. To the best of our knowledge, there are only 7 reported cases of de novo AIH after LT for PBC [4]. De novo AIH can be asymptomatic and manifest as unexplained graft dysfunction or acute in an otherwise stable transplant recipient. Most patients have hypergammaglobulinemia, increase serum IgG levels or autoantibodies. The most common serological markers have been AAN, SMA, or both [2–4]. In some cases, as for our patient, de novo AIH has been diagnosed in the absence of autoantibodies and in some patients without increase serum IgG levels or autoantibodies [5, 6]. In these instances, the diagnosis has depended on liver tissue examination or response to corticosteroid therapy. Portal and periportal hepatitis with lymphocytes and plasma cells constitute the typical histological features of de novo AIH and perivenular cell or bridging centrilobular necrosis may also be present [2–4]. In our patient, the diagnosis of de novo AIH was the most likely in the absence of bile duct damage or loss, excluding PBC recurrence, as well as raised Ig G level and response to corticosteroids. Some studies have applied the comprehensive diagnostic scoring system of the IAIHG to patients with graft dysfunction and presumed de novo AIH but these scoring has not been developed or validated as a discriminative diagnostic index for AIH after LT [4].

The pathogenesis of de novo AIH is not well known but several mechanisms have been implicated. This condition is not necessarily autoimmune, but rather alloimmune. Viral infections of various causes may stimulate an autoimmune response, loss lymphocyte T suppressor-cell function, and molecular mimicry (an initial immune response towards a non self-antigen that became directed toward a structurally similar self-antigen). Our patient showed no antecedents of rejection or viral infections before the diagnosis of de novo AIH. The calcineurin inhibitor used by transplanted patients can interfere with the maturation of T cells and the function of regulatory T cells which resulted in emergence and activation of auto aggressive T cell clones [7]. Some patients with de Novo AIH have antibodies against glutathione-S-tranferase-T1 (GSTT1), a drug metabolizing enzyme that is produced in large quantities in liver and kidney cells. Twenty percent of whites do not carry the gene GSTT1. GSTT1 donor-recipient mismatch and the presence of anti-GSTT1 antibodies might be risk factors for the development of de Novo AIH [8]. The GSTT1 antibody wasn't sought in our patient. De novo AIH must be distinguished from acute rejection, chronic rejection, viral infection and drug toxicity. The histological features and the time interval between disease occurrence and liver transplantation are essential to direct the diagnosis effort [3–9]. Acute rejection usually occurs within the first 30 days after transplantation and it is characterized by portal and central endothelitis, bile duct damage and eosinophils, whereas chronic rejection usually develops 3-12 months after transplantation and it is characterized by cholestatic laboratory findings, bile duct loss affecting more than 50% of the portal tracts, small artery loss, perivenular fibrosis, and foam cell obliterative arteriopathy [3]. Treatment should be based on conventional strategies for AIH. Prednisone alone or combined with azathioprine constitutes the essential therapy and mycophenolate can be considered as an azathioprine replacement [3]. Adults are generally given an initial dose of 30mg Prednisone combined with 1-2mg per kg azathioprine per day [10]. The dose of steroid is reduced according to response to a maintenance dose of 5-10mg daily. In the absence of response, azathioprine should be substituted with mycophenolate mofetil [10]. This strategy is the key for a successful outcome. Switch in the calcineurin inhibitor, treatment with rapamycin and re-transplantation are salvage considerations [3]. In our case the conventional treatment was accompanied by marked improvement in the disease.

## Conclusion

De novo AIH must be considered as a cause of late graft dysfunction in patients transplanted for liver diseases other than AIH, even for PBC. This entity responds to the standard treatment for AIH and this management is graft saving and life saving. Therefore, in the presence of unexplained graft dysfunction, it is important to develop an adequate diagnostic strategy, including determination of serum autoantibodies and liver histology to not miss out the diagnosis of de novo autoimmune hepatitis and to improve the patient outcome.
